# Vaccines as Priming Tools for T Cell Therapy for Epithelial Cancers

**DOI:** 10.3390/cancers13225819

**Published:** 2021-11-19

**Authors:** Lana E. Kandalaft, Alexandre Harari

**Affiliations:** 1 Center of Experimental Therapeutics, Department of Oncology, University Hospital of Lausanne, 1011 Lausanne, Switzerland; 2 Ludwig Institute for Cancer Research, University of Lausanne, 1011 Lausanne, Switzerland

**Keywords:** dendritic cell, vaccines, immunotherapy, epithelial ovarian cancer, personalized treatment

## Abstract

**Simple Summary:**

Despite all of the impressive progress that has been made in the field of cancer therapy, cancer continues to devastate the lives of many. Recent efforts have focused on taking advantage of the patients’ immune system, modifying and employing it to attack cancer cells more efficiently. Therapeutic cancer vaccines are part of the armamentarium used for that purpose. In this review, we discuss the role of the immune system in the fight against cancer, the various strategies that are aimed at engaging the immune system, and how therapeutic cancer vaccines can be used as a self-standing strategy or as a means to leverage other immunotherapies to deliver more efficient results. We elaborate on the obstacles that are present, why immune therapies do not work equally well on all patients, and how vaccines can potentially play a role in improving cancer outcomes.

**Abstract:**

Impressive progress has recently been made in the field of cancer immunotherapy with the adoptive transfer of T cells, a successful personalized strategy, and checkpoint inhibitors (CPI) having extended the survival of numerous patients. However, not all patients have been able to benefit from these innovations. A key determinant of the responsiveness to cancer immunotherapies is the presence of T cells within the tumors. These tumor-infiltrating lymphocytes (TILs) are crucial in controlling tumor growth and their activity is being potentiated by immunotherapies. Although some epithelial cancers are associated with spontaneous T-cell and B-cell responses, which makes them good candidates for immunotherapies, it remains to create strategies that would promote lymphocyte infiltration and enable sustained immune responses in immune-resistant tumors. Therapeutic cancer vaccines hold the potential of being able to render “cold”, poorly infiltrated tumors into “hot” tumors that would be receptive to cellular immunotherapies. In this review, we elaborate on the obstacles that need to be overcome and the strategies that are being explored to that end, including various types of antigen repertoires and different vaccine platforms and combinations with other available treatments.

## 1. Background

Despite significant development in prevention and treatment, cancer continues to affect millions of people worldwide. Approximately 40% of men and women will be diagnosed with cancer at some point during their lifetimes (based on 2015–2017 data) [1]. One out of six deaths in the world is due to cancer, making cancer one of the leading causes of death, second only to cardiovascular diseases [2]. Carcinomas, tumors originating from the epithelium that lines the internal and external surfaces of the body, are the most common type of cancer, accounting for approximately 80 to 90% of all cancer cases. The contribution of the immune system in controlling tumor growth has been thoroughly established [3,4], however, without therapeutic intervention it ultimately fails to prevent death. Nevertheless, by understanding and harnessing the power of the immune system, major progress has been made towards curing cancer. In the last decade, cancer immunotherapy has been a driving force in the treatment of a variety of cancers, including those of epithelial origin.

A slew of immunotherapeutic approaches has been developed with promising results overall. As early as the 1980s, high-dose interleukin-2 (IL-2) was used in metastatic renal cell carcinoma [5,6] and melanoma [7], leading to persistent responses in a fraction of the treated patients. Successful progress continued with the adoptive cell transfer (ACT) of autologous ex vivo expanded TILs. The first demonstration that TILs could mediate tumor regression was in melanoma patients [8,9]. Since then, improvements have been made employing genetic engineering of lymphocytes [10,11], ex vivo stimulation [12,13], and selection of populations with suitable targets [14,15], generating encouraging results in several types of epithelial cancers.

Even more promising results were seen with checkpoint inhibitors (CPI), which are able to induce near-complete sustained responses in a substantial fraction of patients with highly refractory and late-stage cancers [16,17,18,19]. Clinical trials in advanced melanoma patients treated with an antibody against the checkpoint inhibitor (CPI) CTLA-4 (ipilimumab) demonstrated significantly prolonged survival; more than ten years after treatment in some patients [20]. Subsequently, in addition to melanoma, ipilimumab was approved for renal cell carcinoma, colorectal cancer, hepatocellular carcinoma, non-small cell lung cancer, and malignant pleural mesothelioma. Similarly, antibodies against CPI PD-1 or its ligand PD-L1 have delivered exceptional success in treating non-small cell lung carcinoma (NSCLC) [21,22]. To date, four anti-PD-1 antibodies and four anti-PD-L1 antibodies have been approved by the FDA for a wide variety of epithelial cancers and some lymphomas.

Despite these success stories, a significant fraction of patients will not respond to these treatments because the presence of functional T cells within the tumors is a prerequisite for all of these immunotherapies. For example, ACT requires the isolation of T cells from the tumor, while IL-2 and the CPIs target T cells to mediate their effect. However, if tumor-reactive T cells are too rare or do not successfully infiltrate the tumor, inducing the immune system to start an anti-tumor lymphocyte response would be a reasonable approach.

## 2. Control of Tumor Growth by Infiltrating T-Cells

As part of the adaptive immune system, CD8^+^ T lymphocytes play a central role in cancer immunity through their capacity to kill malignant cells upon recognition by the T-cell receptor (TCR) of specific antigenic peptides presented on the surface of target cells. The presence of intratumoral CD8^+^ and CD4^+^ T cells at baseline is independently associated with good clinical outcomes in various types of solid tumors [23,24,25,26]. The immunoscore (CD8 IHC to mark cytotoxic T cells and CD45RO to mark memory T cells) has become one of the biomarkers with prognostic and potentially predictive significance to select patients with the highest likelihood of response to immunotherapy [27].

Beyond the presence of lymphocytes within the tumor, their specificity is also important. Antigens that are uniquely or primarily found within the tumor, and the CD8^+^ T cells that recognize them, have been detected in spontaneously regressing tumors [28,29,30,31], highlighting their significance. Based on these observations, strategies are being developed to engineer CTLs that target these antigens to induce antitumor activity, while avoiding cross-reactivity with normal tissues [9].

Although the immune system is a powerful ally in the fight against cancer, malignant cells can often escape immune recognition. The extent to which the immune system may target a given tumor ranges widely. Some tumors may have very few, or completely lack TILs; these may be referred to as non-T cell inflamed or “cold” tumors. It is not entirely known what determines the infiltration of T cells in the tumor; although, the tumor mutational burden, which typically is associated with increased neoantigen presentation, may play a decisive role in the priming and recruitment of lymphocytes [32,33]. However, even in the presence of abundant antigen, defects in antigen presentation may inhibit the recruitment of T cells.

A hostile tumor microenvironment, usually mediated by diverse immunosuppressive factors including the local secretion of immunosuppressive cytokines, such as transforming growth factor β (TGF-β) and interleukin 10 (IL-10), as well as the downregulation of surface major histocompatibility complex (MHC) class I molecules on malignant cells [34,35,36,37], may be responsible for limiting the efficacy of the antitumor immune response. Other causes include defects in interferon (IFN)-γ signaling [38] and defects of differentiation, migration, and antigen processing by dendritic cells (DCs) [39]. In this context of an immunosuppressive tumor microenvironment, some T-cell inflamed or “hot” tumors may become resistant to the immune response over time.

To generate effective immunotherapy, it is imperative to understand what the underlining cause of immune system failure is for each patient to control tumor growth. For patients who already have functional TILs, ACT is a feasible option, while CPI may facilitate exhausted TILs to control the tumor. Nevertheless, for patients whose immune system does not appear to be primed against the tumor, a multi-step approach will almost certainly be needed for successful results.

## 3. How to Extend Responses in Immunologically Non-Responding “Cold” Tumors

Reversing the immunosuppressive tumor microenvironment poses a significant challenge due to the complexity of the underlying causes; however, for each cause, there may also be an opportunity [40]. When the scarcity of tumor antigen is contributing to the poor immunologic response, demethylating agents, such as DNA methyltransferase inhibitors (DNMTi) and histone deacetylase inhibitors, can enhance the expression of tumor antigens. Endogenous retroviruses [41], silenced Th1 type chemokines, and components of the antigen processing and presenting machinery have also been shown to increase their expression following epigenetic treatment and thus leading to immune response activation [42]. When antigen presentation is inhibited due to MHC-I downregulation, a suitable approach could be to focus on NK cells. Although NK cell immunotherapy remains largely unexplored, the ability of NK cells to target cells lacking MHC-I expression provides a strong rationale for further investigation [43]. A reasonable approach to stimulating tumor infiltration with T cells is to manipulate the tumor endothelium to make it more responsive to T cells. This could be achieved through the selective blockade of known factors, for example, the ET_B_ Receptor that limits T-cell infiltration [44]. Alternatively, chemokines and cytokines could be used to attract T cells within the tumor. This approach has acquired momentum with the intratumoral delivery of these agents. Clinical trials with plasmid IL-12 intratumoral delivery have been promising [45,46]. In addition, intratumoral delivery of IFN-γ was able to stimulate CXCL9, CXCL10, and CXCL11 in melanomas [47]. A few stimulators of IFN genes (STING) agonists are also being used intratumorally in clinical trials [48]. It is expected that the range of agents that can be administered intratumorally will be expanded in the coming years.

Suppression of regulatory T cells (Tregs) is another method of increasing immune activation, and cyclophosphamide has been successfully used for that purpose [49]. In addition, myeloid-derived suppressor cells (MDSCs) are implicated in tumor immunosuppression, blocking their activity by chemotherapeutic agents such as gemcitabine, which appears to induce positive results [50]. Several co-stimulatory receptors are under study; monoclonal antibodies against CD40 [51] and 4-1BB [52,53,54,55] have been developed with agonistic action. A few of these antibodies are currently in clinical trials with promising effects towards inducing T cell infiltration and sensitizing tumors to checkpoint inhibition, albeit with some hepato-toxicities and other side effects.

Similarly, various Toll-like receptor (TLR) agonists are being explored in clinical trials for their ability to stimulate the immune system [56]. TLRs, found primarily on antigen-presenting cells (APCs), recognize danger signals such as conserved pathogen-associated molecular patterns (PAMPs), as well as endogenous damage-associated molecular patterns (DAMPs). PAMPS include glycans, glycoconjugates such as bacterial polysaccharides, flagellin, etc., while DAMPs, also known as alarmins (heat-shock protein, reactive oxygen species, high mobility group protein B1 (HMGB1), calcireticulins, extracellular matrix breakdown products, ATP, DNA, etc.) represent endogenous molecular signals of cell stress or necrosis [57]. Their recognition by the innate immune system (APCs) via the TLR receptors can promote the immune and inflammatory response and activate tumor-specific T cells [58,59].

## 4. Antitumor Immunity through Vaccination

**Figure 1:**
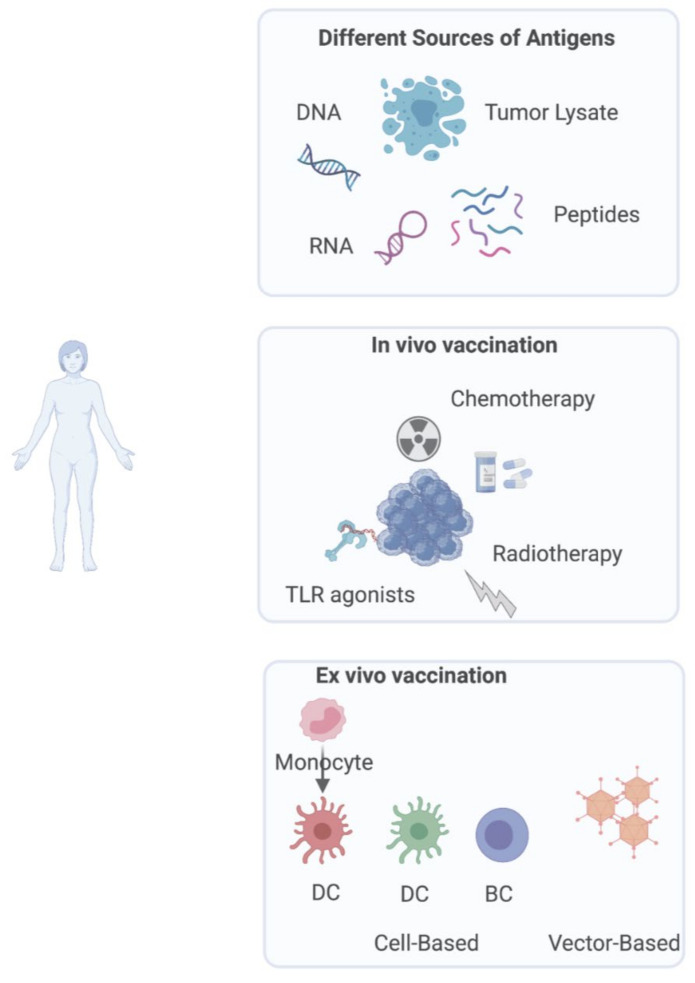
Overview of different sources of antigens and main cancer vaccination approaches. In vivo vaccination targets DCs present in the patient’s body to activate anti-tumor immunity, via a combination of agents (chemo-, radiotherapy and TLR agonists) to potentially elicit both innate and adaptive responses. Ex vivo generated vaccines are either cell-based (dendritic cells or B-cells differentiated ex vivo) or vector-based.

Among the plethora of methods that can be employed to potentiate the immune response against tumors, cancer vaccines have a prominent place. Vaccines are a form of active immunotherapy that aims to induce target-specific activation of the patient’s immune system. In the last few decades, cancer vaccines have acquired a broader sense to include dendritic cell vaccines, in situ vaccination, and vaccination in ex vivo settings, all of which may have a role in the fight against cancer. Regarding in situ vaccination, there is little control of the delivered antigen and no manufacturing platform to be manipulated to adjust the immune response. Alternatively, for classical therapeutic vaccination and dendritic cell vaccination, there are multiple parameters to be considered to optimize efficacy as depicted in Figure 1.

### 4.1. In Situ Vaccination Approaches

Chemotherapy and radiotherapy have not been traditionally considered immunotherapies. On one hand, cytotoxic chemotherapy causes massive death of rapidly proliferating cancer cells but also of immune cells, thus inducing immunosuppression. On the other hand, it has become apparent that chemotherapy, as well as radiotherapy, can induce immunogenic cell death (ICD) in the tumor cells [60]. ICD is a cell death modality that enhances an immune response against dead-cell-associated antigens, which is important when they derive from cancer cells. ICD is a powerful inducer of adaptive immunity, leading to the release of tumor antigens [61] along with DAMPs. Cumulatively, these and the downstream events they trigger, increase antigen uptake and processing and promote DC maturation. These DCs can then migrate to lymph nodes where the priming of T cells specific to tumor antigens takes place. Tumor-specific T cells may subsequently infiltrate the tumor. Following radiotherapy, infiltration of tumor-specific T cells has been described in distal sites (abscopal effect) not directly treated with radiation, highlighting the function of the immune system [62]. In addition, a few chemotherapeutic agents have been shown to have a direct effect on immune cells, such as depleting T regs and MDSCs, further potentiating the immune response. Preclinical studies and clinical trials have been conducted where either chemotherapy or radiotherapy was followed by classic [63,64] or DC vaccines [65], ACT (clinicaltrials.gov: NCT00338377; NCT01585415; and NCT01659151) or CPI [66,67,68], with promising results. However, it is important to note that choosing the right dose and schedule of chemotherapy and radiotherapy may be at least partially to blame in many unsuccessful trials [69,70,71]. A lower dose (for both chemotherapy and radiotherapy) than the standard of care is generally considered more appropriate when in the context of in situ vaccination [72] to increase DC maturation and infiltration of T cells. Non-myeloablative lymphodepleting chemotherapy, in particular, is used prior to ACT to facilitate the grafting of adoptive T cells [73].

Oncolytic viruses can work similarly. Oncolytic viruses naturally, or due to engineering, can target, multiply within, and lyse tumor cells.

As a result, the immune system is primed both by recognizing infected cells and by sensing their ICD. These viruses can be further engineered to minimize their pathogenicity and enhance immunogenicity. To date, talimogene laherparepvec (T-VEC) a herpes simplex virus type 1 is the only oncolytic virus therapy to be approved by the FDA, and clinical trials are ongoing to expand and improve its use [74]. In this context, chemotherapy, radiotherapy, and oncolytic viruses are being employed to deliver antigens and stimulate the immune response, and are therefore considered a type of in situ vaccination that can be combined with other immunotherapies to deliver efficient results. This approach is not only easy to implement since is not labor and technology-intensive, but it also takes advantage of the entire antigenic repertoire of the tumor. However, the efficient delivery of oncolytic viruses poses a challenge; currently the most widely used method is the local intratumoral delivery (versus systemic). Several approaches are being tested to maximize the delivered dose, such as finding the optimal packaging material (polymers, nanoparticles, liposomes, etc.), incorporation of cell-targeting ligands, cell carrier systems, and other methods including ultrasound or magnetic targeting [75,76,77].

### 4.2. Classical Therapeutic Cancer Vaccines

These consist of exogenous administration of tumor antigens along with some adjuvant. The antigen can be administered in the form of DNA, RNA, or peptides [78]. DNA and RNA can act as adjuvants themselves and can be easy to manufacture in sufficient quantities. DNA vaccines are most effective when they are injected intramuscularly in combination with electroporation. An interesting concept that can be applied to DNA vaccines is, in addition to the tumor antigen, to incorporate genetic information to encode proteins that would facilitate the immune response, such as chemokines [79]. RNA vaccines can be injected intramuscularly, directly into the lymph nodes, or they can be administered intravenously as lipoplex nanoparticles [80]. The approval in 2020 of the first mRNA vaccines (against COVID-19) generated significant momentum in mRNA vaccine research that also includes cancer.

Peptide vaccines are usually made of synthetic long peptides (SLP, 25–30 amino acids) that first need to be internalized and processed to be presented by cells. This ensures that only APCs will be able to present relevant antigenic epitopes, which will also provide costimulatory signals to avoid suboptimal activation of T cells due to lack of co-stimulation. The choice of adjuvant to be combined with peptide vaccines is crucial. Peptide-based vaccines alone are poorly immunogenic, therefore they require proper delivery systems combined with adjuvants to be effective. Most frequently, the emulsions-based (oil-in-water, water-in-oil, etc.) or liposome-based systems have been used and studied, but virosomes and polymeric particles have also been explored for peptide delivery [81].

### 4.3. Dendritic Cell Vaccines

Dendritic cells loaded with tumor antigens have been studied for the treatment of cancer for more than two decades [82] and despite the FDA approval of the first APC vaccine in 2010, they still have not delivered on their promise. Nevertheless, they generate a lot of interest and their relatively safe profile encourages continued research. Monocytes are usually isolated from blood and differentiated ex vivo to monocyte-derived DCs. Although this type of DCs may not be the most physiologically relevant, it has been the only feasible option until recently.

A novel approach to use naturally circulating DCs may prove advantageous [83]. DCs can be loaded ex vivo with peptides or they can be transduced to present them. However, these methods require prior knowledge of the targets and the prediction of antigenic sequences to be used. On the contrary, loading the DCs with tumor cell lysates not only is less labor-intensive but also ensures a broad range of antigen presentations that can engage both CD4^+^ and CD8^+^ T cells. Several methods have been developed to process tumors for DC vaccine applications including hypochlorous acid (HOCl) oxidation, freeze-thaw cycles, and UV irradiation. HOCL is a strong bactericidal oxidant, capable of potentiating the immunogenicity of protein antigens to increase their uptake and processing by APCs as well as activation of T cells [84]. Preparation is necessary to eliminate immunosuppressive factors that are found within the tumor and to make it more immunogenic by inducing ICD to some extent. Several routes of administration have been investigated for DC vaccines, with the subcutaneous route being the most common, however, some data suggest that intravenous or intranodal administration may be more effective.

## 5. Choice of Antigen, Personalized Targets

Initial attempts of making cancer vaccines were focused on tumor-associated antigens: self-antigens that are expressed in tumors but also (i) in germline tissues with only a very limited expression in adult tissues (e.g., NY-ESO-1 and MAGE-A3), (ii) in differentiated tissues from which the tumor originates (MART-1 and CD19), and (iii) in normal tissues at a lower expression than that of the tumor (HLA-A*02:01). Many of these antigens are shared across patients and there were hopes of the development of “off-the-shelf” vaccines. Vaccine trials against tumor self-antigens have largely been less than encouraging [85], which may be due to pre-existing immune tolerance. Another class of antigens that has gained prominence in the last decade is tumor neo-antigens or non-self antigens [85,86]. These are derived from non-synonymous mutations that occur during the process of tumorigenesis. Due to their restricted expression in the tumor cells, these may be both more efficient and safer than tumor-associated antigens. However, since the repertoire of mutations (the mutanome) is unique to each patient, a highly personalized approach needs to be taken every time. Importantly, identifying these neoepitopes and predicting cognate MHC binding is a cumbersome task involving genome and transcriptome sequencing and sophisticated predicting algorithms. Once the neoantigen sequences have been identified, they can be used for classical, DC, or ex vivo vaccination. However, the use of tumor lysate for DC loading is also a highly personalized approach with the difference of being target agnostic and thus less labor-intensive. These approaches are depicted in Figure 1.

## 6. Vaccine Priming in Combination with Other T-Cell Based Therapies

**Figure 2:**
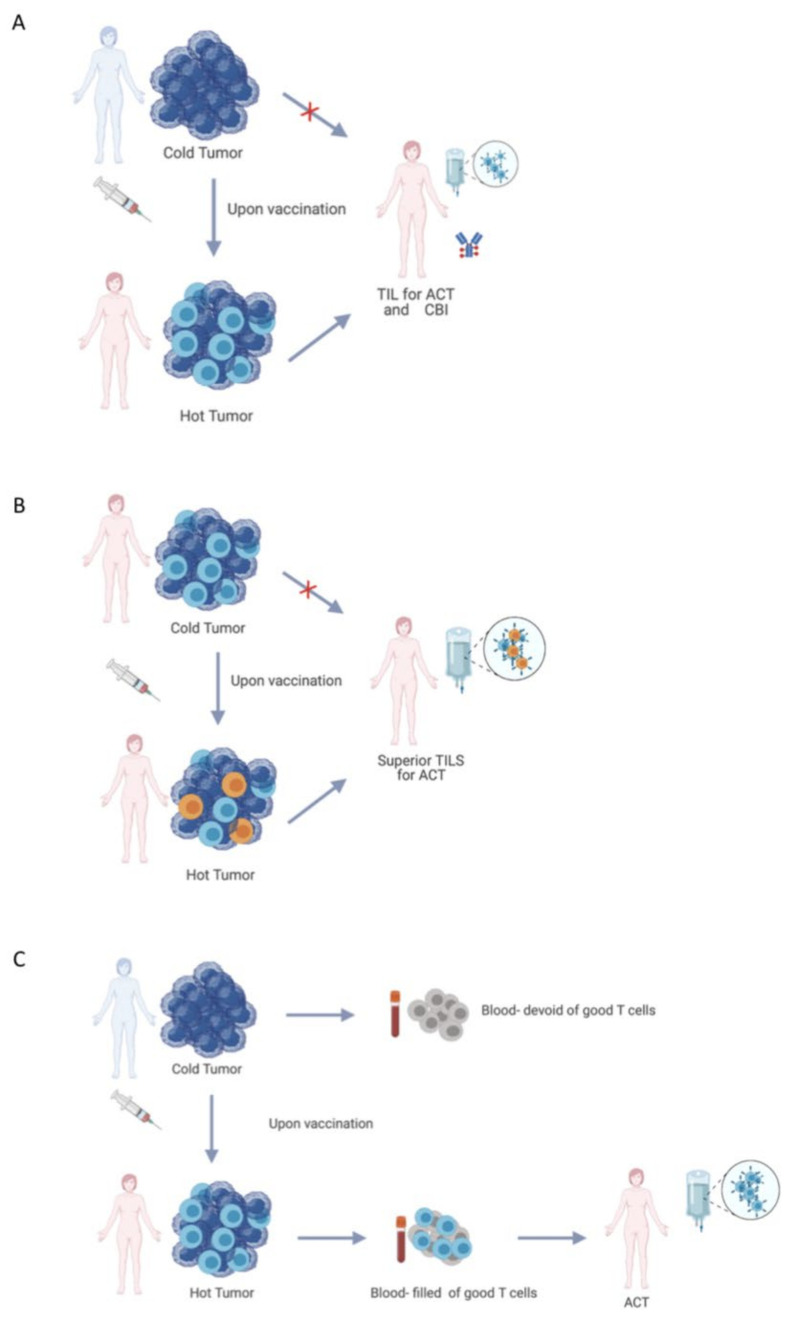
The different applications of vaccination strategies in improving T-cell therapy. (**A**) Immunologically “cold” tumors can be preconditioned by cancer vaccines to attract TILs to the tumor site and make it suitable for adoptive TIL transfer or checkpoint inhibitor therapy; (**B**) The immune response against tumors already containing TILs can be enhanced by priming vaccination to prime the superior TILs; and (**C**) Immunologically “cold” tumors can be turned into “hot” tumors upon vaccination, filling peripheral blood with circulating good T cells which potentially could be used for adoptive T cell therapy.

Despite the significant progress that has been made in the field of therapeutic cancer vaccines, due to the complexity of the disease, it is clear that vaccination as monotherapy is not sufficient. Instead, vaccination can be used as a part of a toolbox of therapies that, in combination with other modalities, will ultimately lead to better outcomes. We highlight in this section some of the proposed methodologies of vaccine utilization to potentiate T cell immunity (Figure 2).

### 6.1. DC Vaccines Combined with ACT

The hypothesis that DC vaccination could improve the efficacy of ACT was first tested in mice with a melanoma expressing glycoprotein 100 (gp100) tumor antigen. Gp100 is a membrane-bound glycoprotein and is a melanocytic differentiation antigen expressed in pigmented cells and most melanomas. Mice were treated with cultured activated T cells engineered to express a T-cell receptor specifically recognizing gp100 with concurrent gp100 peptide-pulsed DC vaccination. The combination of DC vaccination and ACT led to a more robust immune response than either treatment alone [87], paving the way for further research. Similar results were obtained with a different melanoma mouse model, using intratumoral DC vaccination in combination with ACT. Additionally, it was shown that multiple intratumoral vaccine injections could further improve the response [88].

Transferring these studies to humans was a huge development that succeeded for patients with MART-1 positive melanoma. Autologous peripheral blood lymphocytes were engineered with a transgenic MART-1 T cell receptor and administered together with a MART-1 peptide-pulsed DC vaccine. The study turned out to be feasible, but cryopreservation appeared to limit the potency of T cells and the results were rather modest [89]. A later study used TILs that demonstrated reactivity to MART-1. No difference was shown between the groups that received TIL therapy alone or in combination with DC, however, the study was not designed for that differentiation [90].

Despite the lack of striking results, the interest in the TIL and DC combination remains high because of low therapy side effects; in contrast, conditioning for TIL with chemotherapy or radiotherapy and IL-2 administration is associated with severe adverse events. With this under consideration, Poschke and colleagues hypothesized that combining TILs infusion and tumor lysate DC vaccination could circumvent the need for preconditioning and IL-2. Indeed, the scheme was well tolerated and the results were promising, however, the phase I clinical study was not designed for comparison with other treatments [91].

Although it is difficult to obtain clear positive results with most phase I clinical studies, due to their focus on feasibility and safety, a very encouraging study was recently reported in metastatic melanoma patients that had shown resistance to immune checkpoint inhibitors.

The patients received TIL ACT followed by five intradermal DC-vaccine injections. Just as before, the DC vaccine was loaded with autologous tumor lysate. The patients also received cyclophosphamide/fludarabine preconditioning and TIL administration was followed by IL-2. All four treated patients had long-lasting persistency of the injected TILs, two had partial responses, and the other two had complete responses that were still ongoing when the report was published [92].

Positive results have also been obtained with ovarian cancer. In a Phase I trial, vaccines consisting of DC pulsed with autologous whole tumor lysate were used in patients with recurrent ovarian cancer. Next, vaccine-primed lymphocytes were collected from peripheral blood and stimulated with CD3/CD28. The study was deemed feasible and well-tolerated [93]. A similar study was later conducted recapitulating previous results and showed neoantigen-specific T-cell responses and epitope spreading [94]. One particularly interesting aspect of these studies is that the collection of T cells occurred following DC vaccination, therefore, they were primed in vivo to tumor antigens. This differs from the melanoma studies where the ACT and DC vaccination were administered concurrently. Although these studies are not comparable, it is intriguing to consider whether the optimal approach would be to first vaccinate the patients to harvest T cells that are primed to respond to the tumor or to vaccinate after (or concurrently to) ACT to maintain the activity of T cells and to obtain longer-lasting responses. The answer may depend on the immune status of the tumor. It is possible that “cold” tumors may need vaccination first, to ensure a sufficient tumor-specific T cell population. The melanoma and ovarian cancer studies also use different sources of T cells. To the best of our knowledge, there are currently no studies administering a DC-vaccine followed by TIL ACT. However, as we expect that vaccination increases T cell infiltration in the tumor, some tumors may become suitable for TIL preparation only following effective vaccination.

### 6.2. Vaccination and CAR-T Cell Therapy

CAR-T cell therapy has been a tremendous success in the treatment of hematologic malignancies but its efficacy has been limited in solid tumors, in part due to inadequate tumor infiltration and poor functional persistence of CAR-T cells. Several recent studies in mice have explored combining vaccination with CAR-T cell therapy using a variety of different vaccine strategies: (i) a DC vaccine loaded with the corresponding CAR antigen WT1 [95], (ii) a nanoparticulate RNA vaccine designed for body-wide delivery [96], (iii) a Clec9A nano-emulsion selectively targeting and activating Clec9A+ cross-presenting DCs, and (iv) an amphiphile CAR-T ligand designed for lymph node targeting and able to insert itself into plasma membranes [97]. These studies demonstrate that vaccines can increase the efficacy of CAR-T cells as well as the tremendous progress achieved in the field of vaccine biotechnology and material science.

### 6.3. Ex Vivo Vaccination (Manufacturing Setting)

Autologous lymphocytes either isolated from peripheral blood or from within the tumor can be primed ex vivo with tumor antigens or antigen-presenting cells, expanded, and reintroduced to the patients with a greater potential to attack the tumor. There are a few variations of this approach; for example, stimulation of lymphocytes may be done by autologous tumor cell lines or tumor organoids [98]. Alternatively, DCs loaded with antigen, whole-cell lysates, and tumor-specific peptide antigens have also been explored [12]. A few ACT clinical trials are currently underway using ex vivo DC stimulated TILs (clinicaltrails.gov NCT04032847, NCT03997474, and NCT0463574) and their results are eagerly anticipated.

The possibility of using peripheral blood lymphocytes (PBLs) expands the scope of this application, as it can be applied to inoperable tumors, as well as to tumors that do not have sufficient lymphocyte infiltration. In this case, several rounds of stimulation may be needed to achieve sufficient numbers of TILs [13]. Sorting of lymphocytes using various activation markers (CD137, PD-1, etc.) has also been employed to enrich tumor-reactive CTLs [99,100].

### 6.4. Vaccines Combined with CPI

Vaccines can also be combined with CPI therapy to rescue a CPI-resistant phenotype. As discussed earlier, CPI mediates their effect by relieving inhibitory signals on CD8+ T cells; vaccination enables the presence of these cells in greater numbers than they would otherwise be found and potentiates the effect of CPI. Several studies have investigated this synergy between vaccines and CPI. Initial studies were conducted in mouse models and the results were very encouraging in melanoma [101], ovarian, and colon cancer [102,103], as well as glioma [104], prostate [105,106] and pancreatic tumors [107], which are typically poorly immunogenic. For example, in a mouse model with a TC-1 expressing E7 protein derived from HPV, the administration of the anti-E7 cancer vaccine increased PD-1 expression on T cells resulting in concomitant tumor regression. This effect was further enhanced by the addition of a PD-1 blockade, which synergized with the vaccine in eliciting antitumor efficacy [108]. These studies, which involved both PD-1 and CTLA-4 inhibitors, either in combination or individually, and several types of vaccines, paved the way for clinical trials. Checkpoint inhibitors (other than anti-PD-1 and anti-CTLA4) such as anti-NKG2A, have also received interest in combination with vaccines [109].

Several clinical trials have been conducted to combine vaccination with anti-CTLA-4 in melanoma patients using peptide vaccines. All of these trials gave rather modest results [110,111,112]. Other trials with GVAX (irradiated tumor cells secreting GM-CSF) [113], MART-1 peptide-pulsed DCs [114], and DCs electroporated with mRNA of melanoma-associated antigens [115] were more encouraging, showing improved survival and persistent T cell activity. The combination of GVAX with anti-CTLA-4 also improved survival in pancreatic adenocarcinoma patients [116]. Comparably positive results in overall survival were obtained with the combination of PROSTVAC (a poxvirus-based vaccine for prostate cancer) and anti-CTLA-4. An anti-PD-1 plus GX-188E therapeutic DNA vaccine (encoding E7/E7 fusion protein of the human papillomavirus (HPV) subtype 16/18 and the cytokine FLT3-ligand) was evaluated in HPV-16 or -18 positive advanced cervical cancer in a Phase II study (*n* = 40), where the interim analysis showed preliminary antitumor activity and that the combination was safe [117]. FixVac, an intravenously administered liposomal RNA vaccine targeting non-mutated TAAs was tested in advanced melanoma patients in a Phase I study [118]. The interim analysis revealed that FixVac, alone or in combination with anti-PD1, mediated durable objective responses in checkpoint-inhibitor-experienced patients with unresectable melanoma, with clinical responses accompanied by the induction of strong CD4+ and CD8+ T cell-mediated immune response against the vaccine antigens [118]. A few recently published studies of cancer vaccine and anti-PD-1 combination therapies [119,120,121] showed promising outcomes overall, but there are several more clinical trials ongoing and awaiting conclusion.

## 7. Conclusions

Choosing from a wide array of vaccine platforms and a combination of many different therapies requires the meticulous consideration of numerous parameters. Each patient and each cancer is unique. Selecting the right treatment for each patient is of paramount importance and finding the tools to characterize each tumor is an ongoing endeavor. Although we have come a long way in the treatment of cancer and a standard of care (SOC) treatment has been established for every tumor, there are still many treatments that need to be tested thoroughly, and in combination, that can improve the survival and quality of life of cancer patients.

Therapeutic vaccines in cancer have been studied for a couple of decades and although their potential is evident, we have yet to optimize their use. In addition to what has been previously discussed, timing, dosing, and scheduling, all play a role in the effectiveness of cancer vaccines. SOC often leads to immunosuppression, and the vaccines need to be administered at a time when the immune system can adequately respond.

Utilizing different vaccines in ideal boosting approaches may be beneficial, but too many doses can exhaust the T cell population and lead to anergy, while too little may not be enough. Similarly, combining many different approaches may potentially maximize benefits, but it also may lead to the accumulation of toxicities and adverse events, as well as poor compliance.

It has long been apparent that the immune response is a powerful ally in the fight against cancer. For effective long-term disease control, the therapeutic potential of the immune system relies mainly on cancer vaccines with CPI, ACT, and chemotherapeutic agents in a combination of different approaches to optimize antitumor immune responses with the ultimate goal to achieve a cancer cure.
